# Supplementary material for: Plant Metabolic Engineering by Multigene Stacking: Synthesis of Diverse Mogrosides
Source: Int J Mol Sci. 2022 Sep 9;23(18):10422. doi: 10.3390/ijms231810422 (PMC9499096; doi:10.3390/ijms231810422)
Supplement: Supplementary file 1 [file ijms-23-10422-s001.zip › ijms-1881041-supplementary.pdf]

SUPPLEMENTARY INFORMATION

This file contains the Supplementary Figures and Supplementary Tables corresponding to the manuscript: **"Plant metabolic engineering by multigene stacking: synthesis of diverse mogrosides"**

Jingjing Liao[1], Tingyao Liu[3], Lei Xie[2], Changming Mo[4], Xiyang Huang[5], Shengrong Cui[2], Xunli Jia[6] and Zuliang Luo[2*] & Xiaojun Ma[2*]

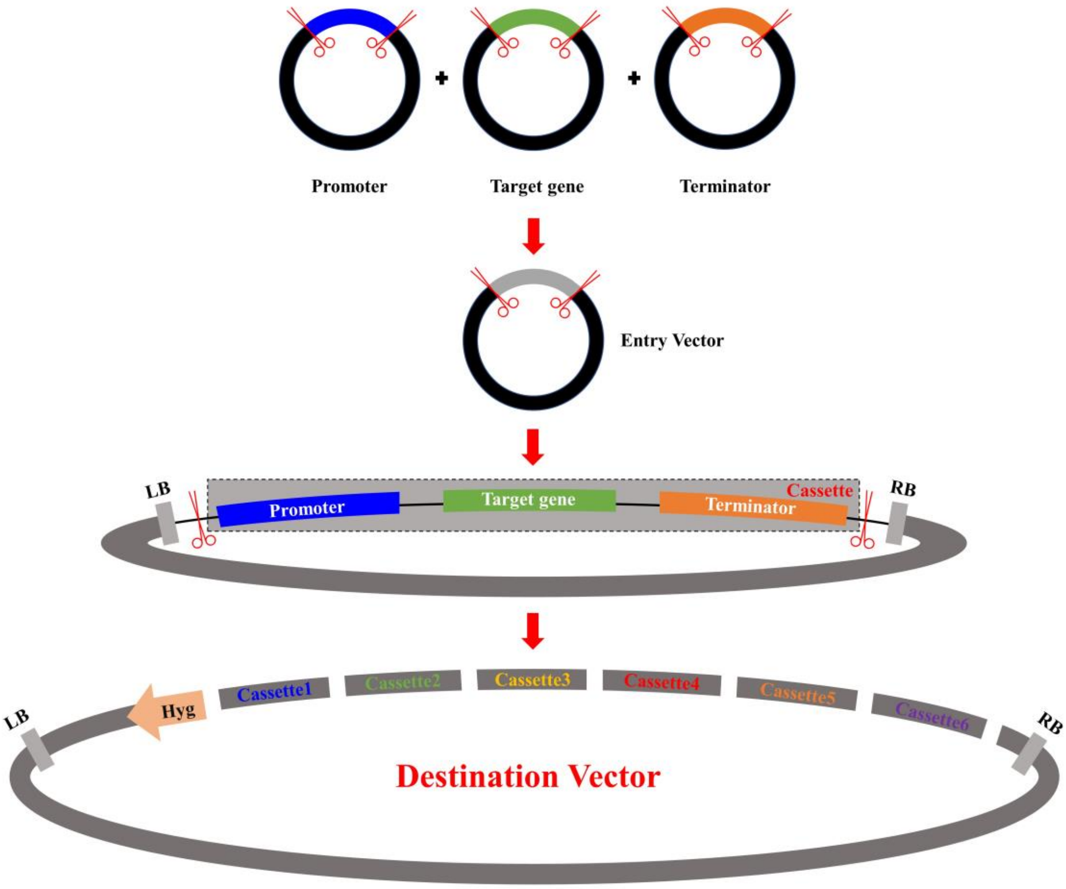

Figure S1. Strategy for multigene stacking. LB and RB are left border and right border, respectively.

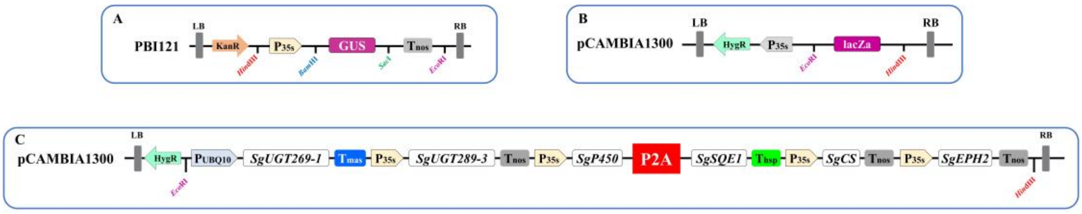

Figure S2. Construction of multigene vector with 6 mogrosides synthase genes in pCAMBIA1300. (A) The map of PBI121 plasmid. (B) The map of pCAMBIA1300 plasmid. (C) The multigene vector U22p-SCE habouring 6 mogrosides synthase genes. The amino acid sequence of P2A is GSGATNFSLLKQAGDVEENPGP. *Bam*HI, *Sac*I, *Hin*dIII and *Eco*RI are restriction enzyme sites.

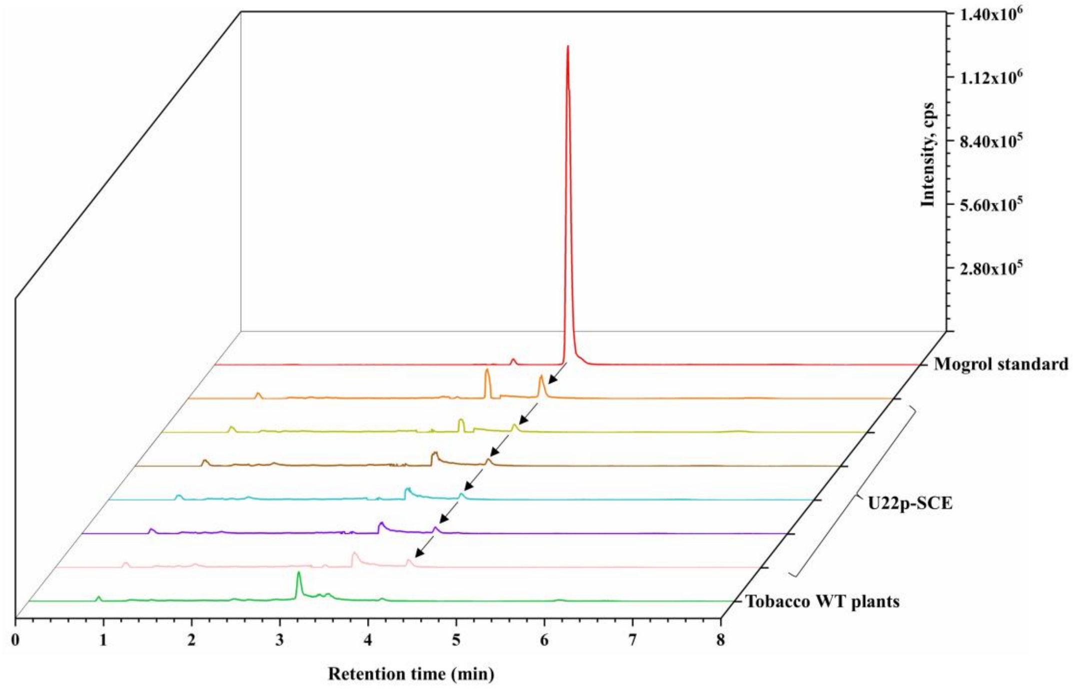

Figure S3. Transient accumulation of mogrol in the leaves of tobacco. Transient total ion chromatograms of mogrol in the leaves of tobacco.

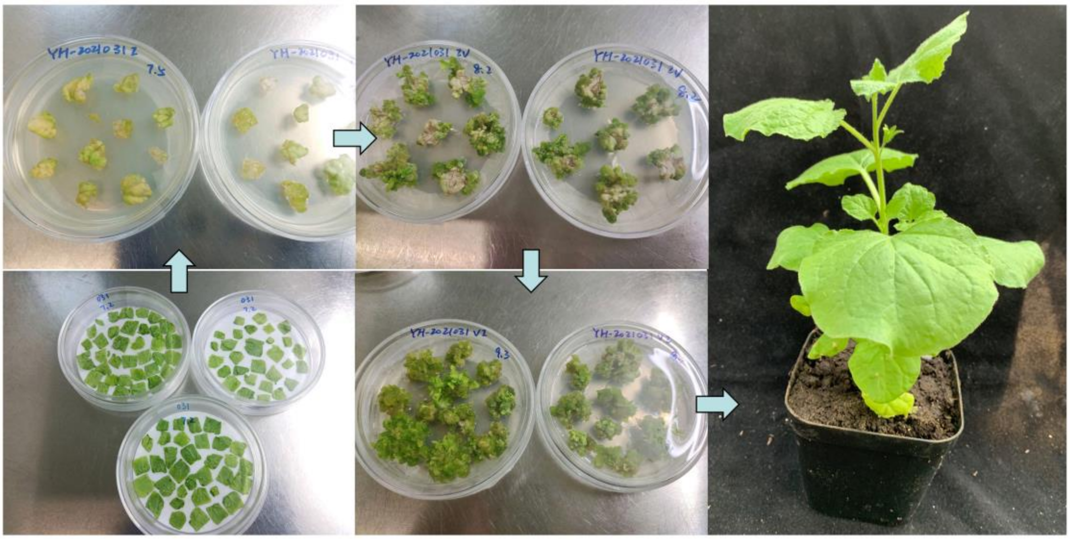

Figure S4. Genetic transformation of tobacco with *Agrobacterium* harbouring the U22p-SCE vector.

Table S1. Primers used for multigene vector construction.

Primers for single gene expression cassette construction.	
**Primer**	**Sequences (5' to 3')**
**PBI121-SgCS-F**	acgggggactctagaggatccATGTGGAGGTTAAAGGTCGGAGC
**PBI121-SgCS-R**	cgatcggggaaattcgagctcTCAAACACTGGTGGACTTCTATAATAAG
**PBI121-SgEPH2-F**	acgggggactctagaggatccATGGAAAACATCGAACACAC
**PBI121-SgEPH2-R**	cgatcggggaaattcgagctcTAACAGAACTTAGAGAAGAAATC
**PBI121-SgP450-F**	acgggggactctagaggatccATGTGGACTGTCGTGCTC
**PBI121-SgP450-R**	cgatcggggaaattcgagctcTCATTCCTTGGGTGTGAACTTCACATGT
**PBI121-SgUGT289-3-F**	acgggggactctagaggatccATGGATGCTGCCCAACAA
**PBI121-SgUGT289-3-R**	cgatcggggaaattcgagctcTCATATTTTAAGCAAGAGAGAAAT
**PBI121-PD7-F**	gaccatgattacgccaagcttATAGGCAACCGTGGACTTCTTCA
**PBI121-PD7-R**	tgatccaccatTTGAGGCTAGGTTTTAGTAGTGAAG
**PD7-SgSQE1-F**	ctagcctcaaATGGTGGATCAGTGCGCGTT
**PD7-SgSQE1-R**	tcttcatcttcatatTTAAACGATTGGCTTAAACAC
**SgSQE1-Thsp-F**	aatcgtttaATATGAAGATGAAGATGAAATATTTG
**SgSQE1-Thsp-R**	aaaacgacggccagtgaattcCTTATCTTTAATCATATTCCATA
**PBI121-UBQ10-F**	gaccatgattacgccaagcttGTCGACGAGTCAGTAATAAACGGC
**PBI121-UBQ10-R**	cgaggttgcaccatCTGTTAATCAGAAAAACTCAGATTAATCG
**UBQ10-SgUGT289-3-F**	taacagATGGTGCAACCTCGGGTACTG
**UBQ10-SgUGT289-3-R**	ccaacatgggagtccaagTTAAAATTTATATGGTTTCAATTTTTTGA
**SgUGT289-3-Tmas-F**	ttttaaCTTGGACTCCCATGTTGGCA
**SgUGT289-3-Tmas-R**	aaaacgacggccagtgaattcGATAATTTATTTGAAAATTCATAAGAAAAGC

[a] The homology arm sequences are noted in red; All restriction enzyme sites are underlined.

**Primers for double-gene expression cassette construction**	
**Primer**	**Sequences (5' to 3')**
**SQE1-2-F**	gaccatgattacgccaagcttATAGGCAACCGTGGACTTCTTCA
**SQE1-2-R**	gtctcaattgccctttCTTATCTTTAATCATATTCCATA
**CS-2-F**	ttactagatcAAAGGGCAATTGAGACTTTTCAA
**CS-2-R**	aaaacgacggccagtgaattcCAGTGAATTCCCGATCTAGTAACATAG
**269-2-F**	gaccatgattacgccaagcttGTCGACGAGTCAGTAATAAACGGC
**269-2-R**	ttcttcttgtcaGATAATTTATTTGAAAATTCATAAGAAAAGC
**289-2-F**	aataaattatcTGACAAGAAGAAAATCTTCGTCAAC
**289-2-R**	aaacgacggccagtgaattcTTAAGTTGGGTAACGCCAGGG

[a] The homology arm sequences are noted in red; All restriction enzyme sites are underlined.

**Primers for triple-gene expression cassette construction**	
**Primer**	**Sequences (5' to 3')**
**SC-3-F**	gaccatgattacgccaagcttATAGGCAACCGTGGACTTCTTCA
**SC-3-R**	gccctttggtcAAAGCGAAAGGAGCGGGC
**EPH-3-F**	tttcgctttGACCAAAGGGCAATTGAGACTT
**EPH-3-R**	aaaacgacggccagtgaattcGTAAAACGACGGCCAGTGAATT
**22-3-F**	gaccatgattacgccaagcttGTCGACGAGTCAGTAATAAACGGC
**22-3-R**	tttgatattttATCTAGTAACATAGATGACACCGCG
**P450-3-F**	gtcatctatgttactagatAAAATATCAAAGATACAGTCTCAGAAGACC
**P450-3-R**	aaaacgacggccagtgaattcCCCAGTCACGACGTTGTAAAACG

[a] The homology arm sequences are noted in red; All restriction enzyme sites are underlined.

**Primers for final gene expression vector construction**	
**Primer**	**Sequences (5' to 3')**
**U22p-4-F**	ctatgacatgattacgaattcGTCGACGAGTCAGTAATAAACGGC
**U22p-4-R**	**tgCTTCAGCAGGCTGAAGTTAGTAGCTCCGCTTCC**CTTATCGTCGTCATCCTTGTAATCTT

**SCE-4-F**	**aacttcagcctgctgaagCAGGCTGGAGACGTGGAGGAGAACCCTGGACCT** ATGGTGGA TCAGTGCGCGTTGG
**SCE-4-R**	acgacggccagtgccaagcttCCGATCTAGTAACATAGATGACACCG

[a] The homology arm sequences are noted in red; All restriction enzyme sites are underlined.

[b] The sequence of P2A polypeptides are noted in green.

Table S2. Primers for PCR detection.

Primers	Sequences (5' to 3')
***SgSQE2*-F**	ATGGTGGATCAGTGCGCG
***SgSQE2*-R**	TCAAACACTGGTGGACTTCTATAATAAG
***SgCS*-F**	ATGTGGAGGTTAAAGGTCGGAGC
***SgCS*-R**	TTATTCAGTCAAAACCCGATGGC
***SgEPH*-F**	ATGGAAAACATCGAACACAC
***SgEPH*-R**	TAACAGAACTTAGAGAAGAAATC
***SgP450*-F**	ATGTGGACTGTCGTGCTCGG
***SgP450*-R**	TCATTCCTTGGGTGTGAACTTCACATGT
***SgUGT269*-F**	ATGGTGCAACCTCGGGTA
***SgUGT269*-R**	TTAAAATTTATATGGTTTCAATTTT
***SgUGT289*-F**	ATGGATGCTGCCCAACAA
***SgUGT289*-R**	TCATATTTTAAGCAAGAGAGAAATTTCA
**Hyg-F**	TTGGCGACCTCGTATTGGGA
**Hyg-R**	CAAGACCTGCCTGAAACCGAA

Table S3. Primers for qRT-PCR analysis.

Primers	Sequences (5' to 3')
***SgSQE1*-qF**	GCTTCGACCATCAACACATTG
***SgSQE1*-qR**	TTCCTCCAAGGCTCAAGTAATC
***SgCS*-qF**	GTTGGGTTGAAGATCCCTACTC
***SgCS*-qR**	CCACAACTGGCTCCCATTAT
***SgCYP450*-qF**	GATGTTCGGTGAGGATGCGA
***SgCYP450*-qR**	TGGTGCCGGGAAAATTCAGT
***SgEPH2*-qF**	AGCTTTGGCTCCATGGTTGA
***SgEPH2*-qR**	CCCAGGTTCTGTCAAAGGCT
***SgUGT269-1*-qF**	CCGATTGAAGTAGCGGAAGAA
***SgUGT269-1*-qR**	CCTCAACGAGCTTCGGTATAAA
***SgUGT289-3*-qF**	CAGAGAAGATGTGCGGAAGAA
***SgUGT289-3*-qR**	TCAGCGACCATCTCATCAAAC
***SgUBQ*-qF**	ATAAAAGACCCAGCACCACATTC
***SgUBQ*-qR**	CCCTTGCCGACTACAACATCC
***NbActin*-qF**	TATTCCCTAGTATTGTTGGC
***NbActin*-qR**	CTGGGGTATTAAAAGTCTCA
***Atactin*-qF**	CACTTGCACCAAGCAGCATGAAGA
***Atactin*-qR**	AATGGAACCACCGATCCAGACAC
